# Load Handling and Repetitive Movements Are Associated with Chronic Low Back Pain among Jute Mill Workers in India

**DOI:** 10.1155/2016/7843216

**Published:** 2016-08-02

**Authors:** S. Goswami, S. Dasgupta, A. Samanta, G. Talukdar, A. Chanda, P. Ray Karmakar, A. Majumdar, D. Bhattacharya, A. Chakrabarti

**Affiliations:** ^1^ESI Institute of Pain Management, ESI Hospital, Sealdah, 301/3A, A. P. C. Road, Kolkata, West Bengal 700009, India; ^2^Department of Mechanical Engineering, Jadavpur University, 188 Raja S. C. Mallick Road, Kolkata 700032, India; ^3^Department of Community Medicine, R. G. Kar Medical College & Hospital, 1 Kshudiram Bose Sarani, Kolkata 700004, India; ^4^The Jute Corporation of India Ltd., 15N Nellie Sengupta Sarani, Kolkata 700087, India; ^5^Regional Occupational Health Centre (Eastern), Indian Council of Medical Research (ICMR), Block DP1, Sector V, Salt Lake, Kolkata 700091, India

## Abstract

*Introduction*. WHO recognizes low back pain as one of the most important ergonomic stressors. Therefore, the present study was designed to find out the magnitude of the problem among jute mill workers in India and identify possible associations.* Methodology*. This cross-sectional workplace based study was conducted among eight (8) selected jute mills of India. Subjects with self-reported back pain for at least last 12 weeks were included and *n* = 717 male jute mill workers actively engaged in work entered the study and completed all assessments.* Results*. Among all participants 55% (*n* = 392) had current chronic low back pain. Age was an important association with subjects in the age group of 40–59 years more likely to have pain (*p* = 0.02, OR 1.44). Regarding ergonomic risk factors lifting of load of more than 20 kg (*p* = 0.04, OR 1.42) and repetitive movements of limbs (*p* = 0.03, OR 0.67) were significant associations of chronic low back pain.* Conclusion*. This study identified a significant prevalence of current chronic low back pain among jute mill workers. Regarding ergonomic risk factors the present study has identified two significant associations: lifting of load above 20 kg and repetitive movements of limbs. Therefore, this study has identified need for workplace interventions in this occupational group employing approximately 3,50,000 workers in India.

## 1. Introduction

Low back pain refers to pain or stiffness in muscle localized below the costal margin and above the inferior gluteal folds, with or without leg pain, and is defined as chronic when it persists for 12 weeks or more [1, 2]. The 2013 Global Burden of Disease Study estimated that globally low back pain is the leading cause of disability with a mean of 72.3 million Years Lived with Disability (YLD) resulting in an approximate 57% increase in YLD from low back pain compared to 1990 [3]. Low back pain has been identified as an important cause of disability among occupational groups and has emerged as an important occupational health issue [4, 5]. World Health Organization (WHO) recognizes low back pain as one of the most important ergonomic stressors and estimates that 37% of all low back pain is of occupational origin resulting in morbidity and work absence with consequent economic loss [6]. Studies from different regions of the world have uniformly identified presence of significant chronic low back pain among manual workers, for example, among manual lifting workers in Thailand [7], among farmers in the US with a 33.2% prevalence [8], in a cohort of male workers in the Swedish construction industry with a 16.5% prevalence [9], and among Chinese workers in a foundry factory of an automobile company [10]. Studies have also identified prolonged bending of trunk, manual handling of heavy objects, lifting from squatting positions, and handling of load as important physical risk factors of chronic low back pain [10, 11].

India has one of the largest work forces in the world and is expected to have problem of chronic low back pain among manual workers with significant economic consequences. Limited studies from India have identified association of significant sickness related absence with low back pain among tannery workers [12]; a 59% prevalence of chronic low back pain among brick kiln workers in rural Southern India [13]; and significant chronic low back pain among female brick workers due to awkward posture, repetitive work, and load handling [14]. However, the problem of chronic low back pain among industrial workers requiring heavy manual work has not been investigated in the Indian context. Jute industry in India with a turnover of approximately Rs. 100 billion (US$ 1.5 billion) employs approximately 3,50,000 workers with 75% of the jute processing units (mills) being located in the state of West Bengal [15]. However, little investigation regarding chronic low back pain has been focused among jute mill workers in India, although such workers encounter heavy physical workload and only one study from India has investigated musculoskeletal disorders among jute mill workers and identified presence of significant musculoskeletal pain lasting many hours after work [16] without specifically investigating chronic low back pain. Considering that an approximate 3,50,000 workers are engaged in jute mills in India the problem of chronic low back pain is likely to have a significant public health impact.

Since inception of the pain clinic at the newly initiated (since 2013) ESI Institute of Pain Management, West Bengal, India, which serves as the referral centre for chronic pain, almost 50%–60% of patients with chronic low back pain were found to be workers from jute mills. In most cases such patients require prolonged treatment resulting in long periods of laying off with consequent economic losses. Therefore, in this context there was a need to investigate the problem of chronic low back pain among jute mill workers and the present study was designed to find out the magnitude of the problem and identify possible associations so that effective prevention and treatment strategies can be developed.

## 2. Subjects and Methods

This cross-sectional workplace based study was conducted in six (6) matched jute mills from West Bengal (WB), with each jute mill from the states of Andhra Pradesh (AP) and Assam. Therefore, participants for the study were selected from a pool of eight (8) jute mills of India. A jute mill is a factory for processing jute. Out of 93 jute mills in India, West Bengal has 70 jute mills, the highest in India, with Andhra Pradesh having 9 mills and Assam two (2) mills [15]. Therefore, jute mills from the states of West Bengal, Andhra Pradesh, and Assam were chosen as sites for this study. Jute mills for participation in the study were chosen by convenience sampling considering ease of access, willingness of the mill owners to participate in the study, and presence of most of the main divisions in the factory for jute processing (namely, jute handling, batching, drawing, spinning, winding, beaming, weaving, and finishing). Workers from following jute mills participated in the study (Table 1). Baseline information for total worker strength for each mill was considered for the year 2013-2014.

For inclusion in the study jute mill workers from the selected mills who are in continuous employment for at least one (1) year and are of 18 completed years of age were eligible for entry in the study. Further, workers currently on active duty in the mills with self-reported back pain for at least last 12 weeks in or near the lumbosacral spine with or without radiating leg pain were considered to be suffering from chronic low back pain (cLBP) and included in the study. Exclusion criteria were presence of pain for less than 3 months and worker currently on sick leave or absent from work or not working continuously for more than one year. Other exclusion criteria were other illnesses with low back pain, for example, infection/malignancy/fracture/metabolic disease/inflammatory disorders and age less than 18 years or above 60 years. All participants were assessed clinically for confirmation of presence or absence of chronic low back pain and ascertained eligibility for inclusion in the study. Inclusion of subjects for the study was carried out by a random screening procedure for presence/absence of chronic low back pain following the employment ID of all available workers on that particular day. Each mill had workers currently on leave due to low back pain, but such workers were not included in the study. Although the intention of the study was to include adequate number of female workers, jute mills mostly employ male workers due to requirement of heavy manual work. Therefore, this study identified only *n* = 14 female workers across all sites and to avoid introduction of bias in analyses exclusively *n* = 717 male jute mill workers from all sites entered the study and completed all assessments.

Data was collected by trained research staffs by face-to-face interview with the selected workers following a predesigned schedule after explaining to the workers the nature, benefits, and risks, if any, of the study and taking their signed informed consent for participation in the study. Information regarding age, family history of musculoskeletal disorders, body weight, height, smoking status and duration in occupation, nature of the job of the worker including ergonomic postures, use of machines and instruments, units of weights lifted, and so forth were recorded on a predesigned schedule. Still and video photographs were taken when considered helpful for the problem analysis and data recording. All questionnaires were validated before use in the study. Following questionnaires constituted the predesigned schedule for assessment of participants in the study.

1. Demographic questionnaire: recorded information on religion, ethnicity, age, gender, marital status, average monthly income, level of education, occupation, and so forth from each participant. This questionnaire is modified from the routine clinical case record form used in the pain clinic of the hospital.
2. Brief Pain Inventory (BPI): long-collected information on pain status at this current time period. BPI assessed average, worst, and least pain intensity at the time of interview as well as over the last 24 hours using 0 (“no pain”) to 10 (“pain as bad as you can imagine”) numeric rating scale. Participants identified location of pain on body map indicating 31 locations. A numeric rating scale from 0 (“does not interfere”) to 10 (“completely interferes”) was used to find out degree at which pain interfered with seven activities (such as general activity, mood, walking ability, sleep, enjoyment of life, normal work, and relations with others) over the past 24 hours [17]. The BPI measures both the intensity of pain and interference of pain in the patient's life along with generating information on pain relief, pain quality, and patient perception about cause of pain. BPI has demonstrated both reliability and validity across different cultures and languages and has been adopted in many countries for clinical pain assessment and in epidemiological studies [18].
3. Ergonomic and workplace questionnaire: adapted from a Chinese study among occupational population and enquired about ergonomic risk factors, namely, load handling categorized by weight, repetitive movements, bending and twisting, standing and sitting, insufficient height or space, use of vibrating tools, and so forth [19].

Information on quality of life of all participants was also collected. The content validity of the instruments was checked by translation to local language (Bengali) and retranslation, as well as review by the investigators of the study. Brief Pain Inventory (BPI) has undergone validation in a number of countries and cultural settings and has also been validated in India [20, 21]. The reliability of the instruments has been ensured by administration of the questionnaires by trained research staff, independent cross-checking of the responses encoded, and random retesting of the questionnaire in approximately 10% of the subjects.

Data collection for estimating parameters based on sample statistic in industries needs to be based on the logic of homogeneity or heterogeneity of the respondents. In most of the industry related studies 90% to 95% confidence level is good enough in most of the situations with an error estimate for the parameter between 5% and 10%. With this assumption the necessary statistical analyses depending on the nature of variables can be conducted to suit the study objectives. In our study we decided to have a 90% confidence interval with an error width within 0.07. With this assumption our *Z*_*α*/2_ (confidence coefficient) = 1.64 and* e* (half the desired interval width) = 0.07/2 = 0.035. Therefore, the required sample size *n* = [(*Z*_*α*/2_)^2^ *p*(1 − *p*)]/*e* ^2^ = [(1.64)^2^(0.35)(0.65)]/(0.035)^2^ = [3.6896 × 0.2275]/0.001225 = 0.8393/0.001225 = 685; that is, approximately *n* = 700.

The study commenced after obtaining necessary approval from competent authority and approval from Institutional Ethics Committee. For ethical reasons, other workers suffering from low back pain, outside the sample chosen for the study, were also examined by the clinician and their treatment was organized at ESI Hospital, Sealdah.

Before analysis all entries were checked and cleaned by ignoring or putting missing value codes for inconsistent or ambiguous values. As variables were mostly categorical and/or ordinal, nonparametric tests were used. Frequency distribution was used to describe demographic variables; participant characteristics; characteristics of pain; workplace characteristics; and quality of life variables. Effects of independent variables on patterns of chronic low back pain and their associations were analyzed using Chi-square test for nonparametric data. Estimate of risk was expressed by Odds Ratio (OR) with 95% confidence interval (CI). Acceptable level of significance was set at *p* < 0.05. Data was analyzed by IBM SPSS Statistics 22.0 (IBM Corporation, New York, USA).

## 3. Results

This study was conducted to find out the magnitude of the problem of chronic low back pain among jute mill workers in India and included *n* = 717 male workers from eight (8) jute mills from three states of India. Almost all workers worked for eight hours/day. The schedule of work each day was split into two halves of four and a half hours and three hours. Most workers were engaged in work for six days/week, that is, 48 hours per week. The main departments in each jute mill included jute handling (processing of raw jute), batching (run spreader machine and soften raw jute), drawing (processing of jute to reduce sliver width and thickness), spinning (process of producing jute yarn), winding (convert yarn to spools and cops), beaming (weaving of jute cloth from yarn), weaving (operation of the loom and producing jute fabric of desired quality), and finishing (improve quality of jute product). Apart from the main departments, a jute mill has supporting departments, for example, maintenance, where workers from main departments are rotated. Mills included in the study had all main departments apart from Assam Co-operative Jute Mill, Assam, which did not have handling and drawing departments.

Almost 47% (*n* = 333) belonged to the age group of 20 to 39 years and the rest were above 40 years of age. In general, the sample had low levels of education with approximately 8% (*n* = 56) completing school education. Only 7% (*n* = 48) workers had a monthly earning of at least INR 12,000 (US$ 182). Almost 58% (*n* = 413) worked for less than 20 years in a jute mill, while the remaining worked for more than 20 years; and almost 51% (*n* = 363) worked for 48 h/week and the rest worked for 40 h/week. Almost 33% had either a parent or a sibling working in a jute mill. More than half of the workers consume tobacco (66%, *n* = 475).

Chronic low back pain was defined as back pain for at least last 12 weeks in or near the lumbosacral spine with or without radiating leg pain. Among the sample of jute mill workers enrolled for the present study, 31% (*n* = 223) were identified to be having current chronic low back pain for at least last 12 weeks. However, 55% (*n* = 392) subjects had current chronic low back pain with or without radiating leg pain for at least past 12 weeks satisfying laid down inclusion criteria for chronic low back pain. There was no difference in prevalence of chronic low back pain among jute mill workers by site, that is, participating jute mills (*χ* ^2^ = 4.30, df = 7, *p* = 0.75). The prevalence of chronic low back pain by department across all jute mills was in handling 56%, *n* = 13, batching 48%, *n* = 34, drawing 55%, *n* = 30, spinning 49%, *n* = 56, winding 52%, *n* = 32, beaming 62%, *n* = 23, weaving 61%, *n* = 134, and finishing 58%, *n* = 49; *n* = 21 workers with chronic low back pain were from supporting departments. Approximately 26% (*n* = 185) of all workers also had a family history of chronic low back pain. Regarding intensity of pain among workers with chronic low back pain during the current week 66% (*n* = 233) had mild pain, 28% (*n* = 100) had moderate pain, and 6% (*n* = 21) had severe pain. When associations of subject characteristics were considered with presence or absence of chronic low back pain, age was identified as an important factor with subjects in the age group of 40–59 years more likely to have pain (*χ* ^2^ = 5.83, df = 1, *p* = 0.02, OR 1.44, 95% CI (1.07–1.93)). Although sanitation emerged as a significant factor, this is unlikely to have a real effect as almost no worker used western system of sanitation. No other participant characteristics had a significant relation with chronic low back pain (Table 2).

Ergonomic risk factors considered for the present study were lifting load above 20 kg, carrying load above 20 kg, pulling load above 20 kg, standing or sitting for long duration, twisting or bending trunk awkwardly, maintaining the same posture for long duration, repetitive movements of limbs, and position of the work station. When associations of ergonomic risk factors were considered with presence or absence of chronic low back pain, lifting of load of more than 20 kg (*χ* ^2^ = 4.02, df = 1, *p* = 0.04, OR 1.42, 95% CI (1.0–2.0)) and repetitive movements of limbs (*χ* ^2^ = 4.92, df = 1, *p* = 0.03, OR 0.67, 95% CI (0.50–1.0)) were identified as significant associations of chronic low back pain. No other workplace characteristic had any association with chronic low back pain (Table 3).

## 4. Discussion

In keeping with pattern of jute industry in India, most mills for the study were chosen from the West Bengal, where maximum numbers of jute mills in India are located. Although the study aimed to recruit both male and female workers, female subjects were few as jute mills mostly employ male workers due to demand of heavy physical work. Similar kind of subject selection has also been observed from other studies on jute mill workers in India [16, 22] as well as Nepal [23].

This study identified a significant 55% prevalence of current chronic low back pain among jute mill workers. Although not investigated among jute mill workers, the problem of chronic low back pain has been investigated in various other occupational settings requiring heavy manual work. Using strict diagnostic criteria for low back pain a South African study among steel industry workers has identified a point prevalence of 35.8% [24]. Among agricultural workers in the American Midwest low back pain was the commonest musculoskeletal pain (33.2%) [4]. Among studies from India tannery workers from Kanpur had high prevalence of low back pain (61%) [12]. The same study also identified that 44% workers had at least one period of sickness related absence over past 12 months. Brick kiln workers from Southern India with heavy physical demand also had a [13] high prevalence of chronic low back pain (59%). Higher prevalence (70%) of low back pain was reported from female brick field workers from West Bengal, India [14]. However, our study did not include jute mill workers who are either on leave or laid-off from job due to chronic low back pain. If the prevalence is considered taking into account such workers, chronic low back pain among jute mill workers is expected to have higher magnitude.

Regarding ergonomic risk factors the present study has identified two significant associations: lifting of load above 20 kg and repetitive movements of limbs. Load carriage (OR = 1.54) and lifting (OR = 4.61) were significant risk factors for low back pain among South African manganese factory workers [11]. Similarly lifting (OR = 2.08) and moving heavy objects (OR = 1.95) were significant risk factors of low back pain among Chinese foundry workers in an automobile factory [10]. Significant association of lifting load above 20 kg (OR = 3.5) has also been reported among tannery workers from Kanpur, India [12]. A study among handloom weavers in West Bengal identified a 68% prevalence of chronic low back pain and also observed that repetitive movements of limbs to operate the looms are a likely risk factor of such pain [25]. Jute mill workers, particularly those engaged in beaming and weaving, employ significant repetitive movements of the limbs and, therefore, such workers are expected to develop chronic low back pain. However other studies also identified other ergonomic risk factors for low back pain, which were not associated with chronic low back pain in the current study, namely, bending heavily with trunk and using the same posture for prolonged periods among Chinese workers [19]; bending trunk heavily among Chinese foundry workers [10]; and prolonged trunk flexion among South African manganese factory workers [11]. Different ergonomic risk factors from different occupational groups from different countries are expected as each kind of occupation requiring heavy physical demand puts different kinds of demand on the musculoskeletal system depending on the nature of work.

The present study is an initial attempt to identify the magnitude of the problem of chronic low back pain among jute mill workers in India. The main strength of the study is to fill a knowledge gap as there is no available information with low back pain among jute mill workers in India where a significant work force is engaged and with low back pain being already identified as a major occupational stressor. Jute mill workers are an important occupational group, particularly in the eastern part of India employing approximately 3,50,000 workers. The study identified a significant 55% prevalence of chronic low back pain in this population with lifting of load above 20 kg and repetitive movements of limbs being significant associations. Therefore, this study has identified need for workplace interventions in this occupational group. Work in jute mills requires prolonged activity with awkward postures with repetitive movements handling heavy loads. Workers are also not conditioned for such musculoskeletal activity and the job requirement also does not permit adequate rest periods. As preventive interventions conditioning of musculoskeletal system for repetitive movement with exercise as well as workstation modification keeping in mind the anthropometric characteristics of the workers can be useful. Further, job rotation through different departments in the workplace and optimum breaks during the work hours can also reduce incidence of chronic low back pain in this population. The observations have also identified the need for a larger study with a higher sample size. Limitations of the study lie in the facts that often there is crossover of workers across different departments in a mill, which makes identification of a particular occupational pattern as possible association difficult. Further, proper definition of sites (mills) by identification of available workplace facilities and recruitment of subjects keeping a balance among departments in a mill would have further improved the quality of observations generated by this study.

## Figures and Tables

**Table 1:** Number of jute mill workers participating in the study from each mill (*n* = 717).

Name of mill	Total number of workers	Number of workers enrolled	% of total workers	% of enrolled workers
Hukumchand, WB	12,031	260	2.0	36.3
Prabartak, WB	2,005	68	3.4	9.5
Shyamnagar, WB	5,032	89	1.8	12.4
Assam Co-operative, Assam	775	58	7.5	8.1
Budge Budge, WB	3,058	52	1.7	7.3
Ludlow, WB	4,639	101	2.2	14.1
Howrah, WB	2,886	49	1.7	6.8
Sri Krishna, AP	2,908	40	1.4	5.5

**Table 2:** Relationship between chronic low back pain (cLBP) and participant characteristics (*n* = 717).

Participant characteristics	With cLBP *n* (%)	Without cLBP *n* (%)	*χ* ^2^ (*p*)
Age			
20–39 years	166 (49.8%)	167 (50.2%)	
40–59 years	226 (58.9%)	158 (41.1%)	*χ* ^2^ = 5.84 (*p* = 0.02)
			OR 1.44 (1.07–1.93)

BMI			
≤25	295 (53.5%)	256 (46.5%)	
>25	97 (58.4%)	69 (41.6%)	*χ* ^2^ = 1.23 (*p* = 0.27)
			OR 1.22 (0.86–1.73)

Education			
Illiterate	53 (57.6%)	39 (42.4%)	
Primary	86 (52.1%)	79 (48.0%)	
Middle school	125 (57.3%)	93 (42.7%)	*χ* ^2^ = 1.87 (*p* = 0.76)
Secondary	97 (52.2%)	89 (47.8%)	
HS & above	31 (55.4%)	25 (44.6%)	

Tobacco			
No	121 (50.0%)	121 (50.0%)	*χ* ^2^ = 3.22 (*p* = 0.07)
Yes	271 (57.1%)	204 (43.0%)	OR 1.33 (0.98–1.81)

Sanitation			
Indian	380 (54.1%)	323 (45.9%)	*χ* ^2^ = 5.55 (*p* = 0.02)
Western	12 (85.7%)	2 (14.3%)	OR 5.10 (1.13–22.95)

Experience			
<20 years	211 (51.1%)	202 (48.9%)	
20 to 40 years	170 (59.4%)	116 (40.6%)	*χ* ^2^ = 5.06 (*p* = 0.08)
>40 years	11 (61.1%)	7 (38.9%)	

Parent working in jute mill			
No	252 (52.9%)	224 (47.1%)	
Yes	140 (58.1%)	101 (41.9%)	*χ* ^2^ = 1.71 (*p* = 0.20)
			OR 1.22 (0.89–1.66)

Working hr per week			
40 hr	189 (52.5%)	171 (47.5%)	
48 hr	213 (57.4%)	158 (42.6%)	*χ* ^2^ = 1.78 (*p* = 0.18)
			OR 1.23 (0.90–1.68)

**Table 3:** Relationship between chronic low back pain and ergonomic characteristics encountered by the participants (*n* = 717).

Ergonomic factors	With cLBP *n* (%)	Without cLBP *n* (%)	*χ* ^2^ (*p*)
Lifting load			
Up to 20 kg	255 (47.5%)	282 (52.5%)	
>20 kg	70 (38.9%)	110 (61.1%)	*χ* ^2^ = 4.02 (*p* = 0.04)
			OR 1.42 (1.0–2.0)

Carrying load			
Up to 20 kg	356 (54.3%)	300 (45.7%)	
>20 kg	36 (59.0%)	25 (41.0%)	*χ* ^2^ = 0.51 (*p* = 0.48)
			OR 1.21 (0.71–2.07)

Pulling load			
Up to 20 kg	317 (54.1%)	269 (45.9%)	
>20 kg	75 (57.3%)	56 (42.7%)	*χ* ^2^ = 0.43 (*p* = 0.51)
			OR 1.13 (0.77–1.67)

Standing long hours			
No	37 (60.7%)	24 (39.3%)	
Yes	355 (54.1%)	301 (45.9%)	*χ* ^2^ = 0.96 (*p* = 0.33)
			OR 0.76 (0.45–1.31)

Sitting long hours			
No	351 (54.3%)	296 (45.7%)	
Yes	41 (58.6%)	29 (41.4%)	*χ* ^2^ = 0.48 (*p* = 0.49)
			OR 1.19 (0.72–1.97)

Twisting of body			
No	126 (52.5%)	114 (47.5%)	
Yes	266 (55.8%)	211 (44.2%)	*χ* ^2^ = 0.69 (*p* = 0.41)
			OR 1.14 (0.83–1.56)

Bending of body			
No	39 (45.9%)	46 (54.1%)	
Yes	353 (55.9%)	279 (44.1%)	*χ* ^2^ = 3.01 (*p* = 0.08)
			OR 1.49 (0.95–2.35)

Prolonged same posture			
No	253 (55.7%)	201 (44.3%)	
Yes	139 (52.9%)	124 (47.1%)	*χ* ^2^ = 0.56 (*p* = 0.46)
			OR 0.89 (0.66–1.21)

Repetitive movements			
No	288 (57.4%)	214 (42.6%)	
Yes	104 (48.4%)	111 (51.6%)	*χ* ^2^ = 4.92 (*p* = 0.03)
			OR 0.67 (0.50–0.96)

Work station position			
Too high	20 (60.6%)	13 (39.4%)	
Too low	156 (57.4%)	116 (42.6%)	*χ* ^2^ = 3.65 (*p* = 0.30)
Away from body	19 (63.3%)	11 (36.7%)	
Others	197 (51.6%)	185 (48.4%)	
